# Henry De Mondeville, the Man and His Writings

**Published:** 1903-02

**Authors:** Charles Greene Cumston

**Affiliations:** Boston, Mass.


### Henry de Mondeville, the Man and his Writings.

**With Translation of Several Chapters of his Works.**

By CHARLES GREENE CUMSTON, M. D., Boston, Mass.

DURING the first half of the middle ages, both medicine and surgery had fallen to a very low level in France, and during this period there is not a single name of any surgeon of note that has been handed down to us. But the traditions of antiquity had been preserved in Italy, where, during the 12th and 13th centuries flourished a number of celebrated schools.

Obliged to leave his country on account of the triumph of a rival political faction, Lanfranc, of Milan, established himself at first in Lyons, where he practised for a certain time, and afterward went to Paris, where his practice and his lectures made him celebrated. He had for a disciple the famous Pitard, surgeon of the King St. Louis, who in his turn was first master and the friend of de Mondeville.

Thus, the traditions of the Italian schools became perpetuated in France by men of genius, and also it must be remembered that de Mondeville went to Bologna, where he studied under the celebrated surgeon Theodoric, Bishop of Cervia, the new methods of dressings which the famous Italian surgeon had been promulgating.

The work of de Mondeville may be considered as a true and complete treatise of general surgical pathology, filled with interesting detail for those desirous of studying the progress and the development of surgery during the middle ages. It is, however, far from my intention to weary the reader with a complete résumé of such a vast work, and I am only desirous of here giving those parts having the most importance for the history of surgery, those where at a glance it will be seen in what way the present is united to the past and how the most magnificent discoveries often in reality have their origin in former times.

The chapter which de Mondeville has written on questions relating to the profession is of particular interest, because it comprises teachings which were totally unknown up to the time of his writing. At the epoch when de Mondeville flourished, there existed several types of surgeons. In the first place, there were the physician-surgeon clerks, which were created by the Schools of Medicine, and among this number may be mentioned William de Salicet, Lanfranc, de Mondeville, and Guy de Chauliac. On this point the old documents are absolutely positive.

Then there was the corporation of laity surgeons, founded after the model of those of other trades, having its masters and its "prudhommes," absolutely independent of the faculty of medicine. The master "chirurgiens jures" on the convocation and presidence of the first surgeon to the King, conferred a license to practise after the candidate had been examined. And, lastly, there were the barbers who practised blood-letting and other small operations which were turned over to them by surgeons belonging to the second class, and who had become physicians. At the commencement of the 16th century they formed the corporation of barber surgeons, which in the 17th century became transformed into the college of "chirurgiens jures."

In de Mondeville's time the physician-surgeons were the only ones possessing an adequate instruction, and for this reason he strongly advises those who wished to practise surgery to first study medicine. If, as it occasionally happened, the practitioners belonging to the second class above mentioned were in possession of a certain skill in operating, their absolute want of rudimentary education and general instruction prevented them from rising above a certain social level. This was much regretted by de Mondeville, and he directed many attacks and sarcasms, accompanied by anecdotes, confirming his very unflattering statements regarding this class of practitioners. And for all this he certainly loved his profession passionately, and placed surgery above all other branches.

De Mondeville says that just as God ordains that surgeons should be honored like other men, so it shows from the acts and the chronicles that the Roman emperors, and the sovereigns who succeeded them, held surgeons in great honor and exempted them from taxation and common servitudes. In these bygone times the surgeons of the palace of the prince were called aliorum examinatores or archiatres.

De Mondeville says that the surgeon desirous of learning his profession should study, listen, discuss, read, and operate, that he should have a subtle intelligence, an excellent natural genius, and strong and agile hands. Since he gives himself up to a very hard and hazardous work, his salary should be greater than other artisans. Unfortunately, in these bygone days bad debts were far greater in number than good ones, just as it exists at the present time, and our author says in this respect that he has not as yet found a man rich enough, or rather sufficiently honest, no matter what his condition might be, to pay what he had promised without being pressed or forced to do so, from which arises the necessity of making arrangements and all kinds of concessions most disagreeable for the surgeon, who as long as he is not paid cannot stop demanding his reward, and should accept neither promise nor guarantee from the patient, but simply a bond or the money.

From these and other remarks that he makes we should not be led to believe that de Mondeville was avaricious or interested. He is of the opinion that a fee or a fixed sum should not be accepted from a friend, but in place of these he accepted victuals and jewelry, cloths, and cups as a sign of old friendship and not as a salary.

As to the poor, the true poor, because already at this epoch rich people came to consult the surgeon arrayed in old clothes in order to pay a smaller fee, de Mondeville never desired a retribution, he operated conscientiously for charity. But he is of the opinion that our art should not be lowered by demanding ridiculously small fees, and he believes that it is better to ask nothing than to allow one's self to be guided by the wish of the client.

In those days the practice of operating was particularly ungrateful, and even dangerous, not alone for the patient. The surgeon was legally responsible for the results he obtained, and in order to cover himself he obliged the patient to give his word that he would in no way do him harm in case the operation was unsuccessful, or else he would obtain the assent of people of rank who possessed authority in the city where he practised.

It was, however, in some cases exposing one's self to death in case of an unsuccessful result, and it is well known how King John, of Bohemia, had a French physician sewed up in a sack and thrown into the Oder because he could not restore his sight to him. And, lastly, at the time when de Mondeville practised everybody undertook the healing art, and this is why he says that ignorant and deceitful persons make fortunes and acquire brilliant reputations, while men of knowledge and who are frank and ripe in experience, miserably live. But it is more than astonishing, it is absurd, not only that these quacks and empirics, but kings, princes, dukes, prelates, and other authorities of the Church, nobles and bourgeois, mix themselves up in the science of dangerous surgical cures, and more especially in the treatment of diseases of the eye, which is very difficult and deceiving, to such a point that it is rarely that one finds a surgeon who is really an expert in these matters.

During the last few years the history of military surgery in the middle ages has interested the curiosity of medical historians, and has been the source of many researches in this direction, but unfortunately the documents left us are few in number and Guy de Chauliac, who gives a résumé of the writings of most of his predecessors, was never present at a single battle. The appearance of de Mondeville's book has thrown much new light on this very interesting subject, because as he says himself he was for a long time attached as surgeon to the person of the king, and to Count Charles de Valois in their expeditions against the English and the Flamands, and was thus able to write a very curious chapter on the extraction of missiles founded on his own experience, and which may be considered as a manual of military surgery of the time in which he lived. Darts, arrows, javelins, lances, and many other weapons were in use in those days, and the wounds produced by them were extremely varied, likewise the operative indications in different cases.

Towards the end of the 13th and at the commencement of the 14th centuries, the cavaliers wore thick armor covering them from their head to their feet, which protected them quite efficiently against blows from swords and many other arms, but the armor was inefficient against sharp arrows which were fired with considerable precision by the archers. The armor was not proof against lances, and the wounds produced by them were very numerous and varied in nature according to whether the point did or did not adhere to the armor which it had pierced through, and likewise that part of the body which it entered.

The surgeon with the aid of a blacksmith was first obliged to remove the armor and employed very ingenious instruments for the extraction of these foreign bodies, usually in the form of pincers. Here is the operative technique as described by de Mondeville. The member which had been wounded by the arrow was tightly bound to a strong bit of timber and then the cord of a strong bow was put on the stretch as if to fire an arrow. The projecting end of the foreign body to be withdrawn was solidly fixed to the cord, which was then released, and de Mondeville says that he has seen this method fail but once. He also relates the case of a wounded man in whom an arrow had completely traversed the lower end of the femur at the level of the condyles. The foreign body could only be removed by hitting one of its extremities with an iron hammer after the limb had been firmly held on a column of wood, which had been hewn out so as to leave a space under the point where the instrument was driven out.

Usually speaking, the foreign bodies were removed by the opening through which they entered, but when they penetrated through the entire limb, or very deeply into it, they were withdrawn at the opposite side from which they entered after an incision had been made over them. The arrows used by the English were small and barbed, which rendered their extraction extremely difficult.

As I have already said, the work of de Mondeville contains many personal ideas and original thought on numerous points that he preached, and opinions are even found which would seem to belong to writers who had lived at a much later date. Thus the famous saying of Ambroise Pare, "Je le pansai, Dieu le guerit," is to be found almost textually when speaking of the extraction of foreign bodies plunged in the various viscera, which may be followed by immediate death, and also where abstaining from their removal will occasionally bring about unexpected cures.

But it is certainly by his method of dressing wounds that de Mondeville is deserving of a special place in the history of surgical science, because he may be considered as the true precursor of the modern antiseptic methods. Contrary to the doctrines taught in the schools of medicine, de Mondeville far from considering suppuration as a necessity, or a salutary vent, believes on the contrary that it is a very dangerous complication and one to be avoided at any cost. The method of dressing wounds that he advises, and which his former master, Theodoric, had inspired in him, may be summed up in the six following propositions: (1) sounds should not be inserted into wounds; (2) bones which compress or wound the surrounding structures must be removed and with violence if necessary, and likewise all foreign bodies which are found between the borders of the wound; (3) the borders of the wound should be united as far as possible; (4) the use of sutures is indicated for this purpose; (5) the wound should be fomented with hot wine and dried with sponges; (6) a plaster of his own invention should then be applied, spread out on a piece of cloth and covered with ordinary compresses wrung out in hot wine over which a bandage is applied. De Mondeville also advises the use of linen compresses in the dressing of wounds, which should not be renewed too often. We have here the practice of antiseptics of the first order, and also the occlusion of wounds as done at the present time. Abstain from passing sounds into the wounds, to wash them with hot wine, and then endeavor to obtain immediate union by sutures, and dressed so as to avoid the contact of the air, is certainly most extraordinary when we realise that these precepts were taught in 1306.

Unfortunately, these wise counsels were quickly forgotten, and fifty years later Guy de Chauliac, who held the scepter of surgery in the Occident, brought back the use of the old methods and the useful part played by the suppuration of wounds was again proclaimed. Shortly after de Chauliac, a very bold and practically unknown Italian surgeon, by name Petri de Largelata, gave distinct rules for the use of the drainage of wounds which he obtained by using the quills of the goose, and which was a sure proof of the fear inspired in him by the presence of pus in the tissues.

Nevertheless the use of antiseptic materials, which far antedated the writings of de Mondeville, still remained in practice and it is quite sufficient to read the works published at this epoch in order to convince one's self of many substances used for dressing wounds.

Although abandoning the precepts given by Theodoric, a certain amount of surgical antisepsis was practised without the knowledge of those who used it, and up to the time of the great wars of the Revolution, and of the Empire, when the simple dressing was advocated, that is to say, an infectious dressing of the highest quality, the use of balsams and aromatics were had recourse to for the greater number of wounds and operations. At the commencement of the 19th century the precepts of the school of Broussais enriched still more those of the surgeons of the "grande armee" and of the Faculty of Medicine. The dressings with serate and charpie reigned without contest, and since this time and up to our days purulent infection and septicemia have raged.

The surgeons of the middle ages attached very great importance to the virtues of compound wines, tinctures, balsams and plasters, in the dressing of wounds, and consequently all their treatises on surgery contain what was then termed an antidotarian, which was simply a manual of materia medica destined to indicate the composition and preparation of these substances. The one contained in de Mondeville's works is particularly interesting concerning the history of drugs and plants of this epoch, and on account of its great exactitude and the detail I will further on in this article give a literal translation of two chapters from it.

The surgeons of the middle ages also gave potions which they considered very useful for the cicatrisation of operative or other wounds, and their formulæ were usually very complicated. De Mondeville was one of the first to doubt their efficacy and advised against their use.

Embalming entered into the attributes of the surgeon and appears to have been a means of considerable emolument for them. De Mondeville enters into very curious details concerning this subject, which aptly demonstrates to what a degree the endeavor was made at this time to preserve bodies. The ordinary methods were essentially defective because with few exceptions the abdomen was not open and the viscera were not disinfected, and it is not at all surprising that bodies thus prepared were not preserved like the Egyptian mummies, and that the tombs of the middle ages now only contain dust and débris of bones.

It is a fact that de Mondeville only enjoyed a relative celebrity among medical writers in the following centuries, and it is only by the work of Gui de Chauliac, who was his disciple, that de Mondeville's name has been handed down to us.

When printing was invented the writings of the older surgeons were published with a kind of passion, Gui de Chauliac in 1478, Lanfranc in 1490, William de Salicet in 1492, Bernard de Gordon in 1495, etc., most of which were published at Lyons. Alone, Henri de Mondeville remained in the dust of the libraries, and his name from this time was hidden in the most profound obscurity.

What we know of de Mondeville has only been obtained by what he has said of himself in his writings. His name is even written in varied manners in the different manuscripts. The French translation from the Latin, which is to be found in the National Library at Paris, calls him Mondeville in one paragraph, while in another it is Esmondeville. The Latin manuscripts give it as Mondavilla, or Mundavilla, Esmondavilla or Esmundavilla, Amondavilla or Amundavilla, Amandavilla, Mandavilla, Armandavilla, Hermondavilla etc., in which the termination of villa or ville, which is so frequently met with in the names of towns in Normandy, would lead to the natural conclusion that the subject of this sketch was a Norman. The greater part of these localities are to be found in Normandy.

The names most frequently met with in the manuscripts are Mondavalla, Mondeville, Esmondavilla and Esmondeville, at the present time called Emondaville. The Dictionnaire geographique et administratif de la France gives an Emondeville in la Manche in the arrondissement of Valognes, and a Mondeville in Calvados, arrondissement of Caen.

Master Henri de Mondeville was in all probability from one of these Norman villages, and he often mentions Normandy in his writings, either for the purpose of recalling names given to certain diseases in the language of this province, or for the purpose of recalling facts that he had himself seen, as for example, the cure of hydrophobia by plunging the patient into the sea. In some instances he gives the Norman form to the French words that he employs.

The date of birth of de Mondeville is unknown, but he died at Paris between 1317 and 1320. After having visited the celebrated cities of Italy, de Mondeville came to study medicine at Montpellier and surgery at Paris, and he made such progress in both these sciences that he soon received the degree of Master.[1]

He professed in both universities with such success that the lecture rooms were crowded with students, the nobility and foreigners from all parts of Europe.

Quesnay says that in order to better introduce himself to the world, de Mondeville appeared to walk in the footsteps of Theodoric and Lanfranc, but that his taste was not that of a simple imitator. Being entirely free from the prejudices which so often lower the mind from authority, he himself judged of his masters, or at least he submitted them to a single judge who could decide their true merit, that is to say, to reason enlightened by experience. Those precepts which had been written and regarded as laws he brought down to their proper principles; he looked for the truth or for the confirmation in the diseases themselves, and not in the works and reputations of writers. The public, which is not always blind to medicine and surgery, was carried away, so to speak, by his singular merit, and Mondeville found in this public confidence an unusual reward. After his death his teachings remained for a long time as a guide to practice, and Gui de Chauliac, who quotes Mondeville 96 times in his works, places him among the greatest masters of his art.

Nicaise says that Mondeville appears to him to have an ardent mind, well nourished by much reading in letters and philosophy, as well as surgery. His honesty is evident, he speaks frankly, and no one escapes his criticism, not even the king.

Mondeville was the surgeon to two kings of France, Philippe le Bel (1285-1314), and his son Louis le Hutin (1314-1316), whose body he embalmed. He professed both at Montpellier and at Paris, and as I have already indicated, his lectures were given with great éclat. He was in France, with his master, Jean Pitard, the surgeon to St. Louis and of Philippe le Bel, the most illustrious representative of surgery at the end of the 13th and the commencement of the 14th century.

For the time in which he lived de Mondeville was a sage, occupying a high position, the highest that could be attained in surgery at that time. He was also a distinguished professor and up

1. Note.—Since he professed surgery in the Faculty of Montpellier and later was in Paris as physician to Philippe le Bel, he consequently must have practised both physic and surgery. He must have received the degree of M. D., otherwise he could not have professed in the University of Montpellier, and there is no doubt but that he practised surgery at Paris, since his name appears in the Index Funebreus Chirurgorum Parisiensinum, ab anno 1315 ad annum 1529.

to the epoch of Gui de Chauliac in 1365, his surgery was the best compilation, the best manual, and the best regulated of any that had existed. It should also be remembered that it was the first work written by a French surgeon.

De Mondeville's style is not as dry as might be expected in a didactic work. Although using scholastic Latin, the universal tongue of the middle ages, he gives from time to time specimens of his caustic humor, especially directed against women in his quality of a bachelor, and he does not use them lightly. For example, he reproaches the ladies of Montpellier of lacing their breasts too tightly, and not enough the rest of their body, and even in the study of anatomy he finds the opportunity of maliciously attacking them, an example of which I extract from his treatise:

Et fuit huic membro hoc nomen veretrum impositum ab hominibus sicut patet per modum loquendi Haly supra tegni tract. De causis cap. 37 dicentis: vidi virum qui habebat veretium, testiculos et vulvam. Sed virga et membrum sunt nomina imposita huic membro a mulieribus per excellentiam sicut patet per modum loquendi earum et causa.

In spite of his professional gravity, he occasionally found a time for jesting, as will be seen by these quotations, as well as other paragraphs in his writings.

Mondeville had a high idea of his profession and placed it even above medicine. His desire was that the surgeon should be at the same time a physician.

Surgery in the 14th century was only a manual avocation, and the surgeons were merely at the beck and call of the physicians, and it must be admitted that the large proportion of the former were ignorant and illiterate. Surgeons cultivated in letters, as was de Mondeville, were the exception, and although they endeavored to suppress charlatans and other quacks, the people and even the nobility continued to place their confidence in the latter class of practitioner. I here give a short extract regarding this matter from the French translation of the Latin of de Mondeville's works, the manuscript of which is preserved in the National Library at Paris:

Toutevoies je me met pas hors du tout en tout ceux qui ne sont pas letrés (que ceste oeuvre profite) ou non. Je di que il est aucuns d'iceus, aussi comme ydiotes, simples et ignorans, et sont merveilleusement orgueilleus et despiteux en cuer, disans que il ont l'oeuvre de cyrurgie, malgre les clers cyrurgiens, de lor parens et de leur predecesseurs et de si lonc temps que il n'en est memoire et dient que il ont d'oir en oir, aussi comme de heritage et de nature; et les croient les lais de ce que il dient, aussi conne parchonniers et compaignons de lor folie; et ensurquetout es jours d'ore les nobles et les princes les croient et par eulz tot le pueple, dont il avient mout de fois griès et maladies perilleuses et aucune fois mort; pour la quel chose a tieux orgueillous qui ne sont pas letrés et se dient cyrurgiens nostre devant dite doctrine ne soit de rien aidant ne a leur paciens, ne a ceux qui les croient, tout aussi comme Dieu ne secourt pas ceux qui l'ont en desdaing.

Or sont autres cyrurgiens, qui ne sont pas letrés qui ne sont pas rebelles et sont plus familiers et se duellent outre maniere que il n'ont conneu la science des letrés en l'art de cyrurgie et recognoissent bien que tel petit de science que il puent avoir aquis, que il l'ont eue des mires et des cyrurgiens letrés. A ceus nostre doctrine soit otroiee et soit profitable a lor salut tant pour eulz comme pour leur paciens en leur maladies; tout aussi comme Dieu ne deneieroit pas pardon a cil qui li requerroit humblement.

The state of surgical science was quite as miserable as its practice, and the Arabs and the Arabists were the principal sources of instruction. Experience was a thing then unknown and was replaced by syllogism to the extreme, and where observation should have come in play all that was done was to quote other writers. The subtilities of dialectic took the place of observation and the most unheard of reasonings explained the natural phenomena.

Surgery had, however, been raised out of this miserable condition in Italy, first by the School of Salerno, and then in the 13th century by that of Bologna. Such treatises on surgery from the pen of Brunus Longobardicus (1252), Theodoric de Lucques, Archbishop of Cervia (1265), Guillaume de Salicet (1275) and that of Lanfranc, of Milan (1296), all of the School of Bologna, served de Mondeville as models for his work, especially that of Theodoric for his treatise on wounds and ulcers and that of Lanfranc for the remainder.

In point of fact de Mondeville was not an original writer, nor was he one of these imitators which advance science. As he himself says in his preface he proposes to write his work on surgery in five treatises, the first of which taken from Avicenna comprised anatomy for the use of surgeons. The second treatise takes up wounds and ulcers after Theodoric; the three other treatises were borrowed from Lanfranc.

The third treatise comprised all diseases *a capite ad calcem*, excepting fractures and dislocations which were to form a fourth treatise. This fourth treatise was never written by de Mondeville because death overtook him before he had even finished the third treatise. But before this he had had time to write the fifth treatise, called the Antidotarium, that is to say, a collection of medicines and remedies such as would be called in later years a surgical pharmacopeia. This division is about the same as that found in the preceding surgeries; among others the one by Lanfranc, and sixty years later Gui de Chauliac adopted no other.

The first two treatises on surgery by de Mondeville were in the first place published from 1306 to 1312, and they form, so to speak, the first edition; the first only differs from the later editions by a few unimportant corrections. This first edition is represented by three manuscripts, one of which is to be found at the National Library at Paris.

The second edition comprises all that de Mondeville had written of his surgery—namely, the first treatise is on anatomy, the second on wounds and ulcers, the third on special diseases, while the fifth, the antidotarium, is represented by four manuscripts, all of which are preserved at the National Library at Paris.

As this latter work of de Mondeville's is a very excellent specimen of its kind in those days, I will here give a literal translation of the second and third chapters of this work, which I believe to be the first ever produced in English. The second treats of the "Repercussiva," while the third deals with the "Resolutiva."

### Chapter II.

This chapter is divided into three parts: (1) special preliminary remarks to introduce the student to the subject; (2) the medicines; (3) explanations.

(1) Avicenna in his Treatise on the Effects of the Different Medicines (Book 2, Section 1, Chapter IV.) says: The repercutive medicine acts in a directly opposite manner to that of an attracting medicine. It cools and densifies the member to which it is applied from its coldness, blunts its attracting heat, contracts its pores, hardens and densifies in one respect the humors which are flowing to the member so that they are not received into it, in the other respect it hardens and densifies the member itself, so that it prevents the humors from entering into the limb. This, for instance, is the effect of solatrum, and Serapio agrees on this point, saying in his Aggregationes, in the fourth sermon: On the

Division of the Effect of said Remedies, "the repercussiva must be cold, of a dense or styptic quality, in order that they may energetically repel." He says also, that besides that there are several kinds of repercussiva, the cold and moist, the non-styptic, the cold styptic, the tonifying, the densifying, the contracting, the expressive, and the opilative. The properties of the various repercussiva are described by Avicenna and Serapio, in the chapters referred to. The cold and moist repercussivum is designated in the above extract from the latter mentioned author, as "inspissative," in contrast to the "rarifying." It contracts the ducts and small pores, but not in every direction as due to these styptic repercussiva. The styptic forms contract the openings of the veins, they are earthy, dense, cold, and have an acrid, but not pungent taste; owing to their density they remain adherent on the outside and cannot penetrate within the contracted pores, and therefore are excluded from the depth of the tissues, but contract the structures by means of their coldness and densify and tonify them.

Serapio calls them impulsive, because they propel humors which they encounter into the interior of the body. The tonifying remedies have the property of toning up the composition and condition of the anatomical structures and modify them in such a manner that the limb will not receive the excessive affluent matter or injurious substance. Some of them effect this by the virtue of an inherent property, such as seal earth and theriac; others, like oil of rose, by virtue of their composition. The densifying remedies condense the humor which they encounter by hardening and densifying it. The contractiva contract the member and render it dense. The expressiva compress the member and thus expel the humors, just as the wine is pressed out of the grape by the press. The opilativa block up the pores and ducts in the member to which they are applied because of the siccative and coagulating properties they possess.

The attractiva act in a directly opposite manner from that of the repercussiva, and since this latter class of remedies have not as yet been treated, I will mention on account of their importance, that they by means of their heat and their subtle qualities attract the humors from the depth of the tissues to the point of application of the remedy. This quality proves to be of great use in ischias, after a purgative has been administered. In the same manner on account of their qualities the remedies remove thorns, arrows, etc., thus castoreum, for example, acts.

From what has here been stated it plainly emanates that all true repercussiva are cold, and it also emanates at the same time how and where they act, that is, only in hot materiæ, for a cold materia is never actually in the true meaning of the word repercussed (driven back), even if it is devoured and destroyed by those remedies which possess some heat, so that the hot remedies, because of that similarity are also called in a certain sense, but only because of a certain misnomer in the expression, repercussiva, which however does not correspond to facts and to the actual condition, therefore only the cold remedies are designated as actual and true repercussiva in contrast to the hot ones. The cold remedies only act against the circulating hot materiæ, and all flow of the humors from limb to limb.

The hot remedies are indicated when cold materia is circulating.

The cold repercussiva are of two kinds; one kind represents the repercussiva in the true and strict meaning of the word, the other in a less strict, wider and incorrect sense.

The former force the approaching materia from the outside inwardly and are appropriately prescribed where the amount and quality of the matter create danger rather than any sharp heat produced by the matter, as is the case with bloody materia, which the experienced surgeon will be able to diagnosticate by palpating with the hand, inspecting it with the eye, as well as from the history obtained from the patient. Now these remedies are in part moist and in part dry, but all of them are styptic in nature.

The second kind, those incorrectly termed repercussiva, are all moist, they never drive back within the tissues any matter which is being discharged outwardly, they merely cool and densify this matter which is flowing to the limb and the member itself, so that after a time no matter is absorbed. They are only indicated when the burning and intense heat are of more danger to the patient than the quantity of the matter present. Such for instance is the case when the matter is purely of bile.

(2) Of these two kinds of remedies, the true and the improperly called repercussiva, some are simple while others are compound. The true styptic repercussiva are the following: solatrum, crassula major et minor, portulao, virga pastoris, psilium, hyoscyamus, hedera, acedula, scariola, the water lily, plantago major et minor domestica et silvestris, the leaves and buds of the unripe fruit of the oak, pear tree, quince, ash, cherry, plum tree, the grape vine, the wild rose, the willow, the poplar, the reed, the asp, rushes and similar things, barley, wheat, oats, lolium, sumach (an Arabian name for a kind of bark used for tanning), barberries blueberries, sour grapes, thorn apple, psidia balaustea, roses, anthera, Armenian clay, cacyimia (a late Latin expression for cadmia and other impure zinc oxydes), potter's clay, nerda ferri, coral, antimony, mandrake, verbena, hepatica, corrigiola, pepper, umbilicus Veneris, maidenhair, sempeviva, hypocisten-grass, bean juice, gratia dei, and also all juices, waters, and oils, as well as the substances themselves and all other extracts that can be made therefrom.

The simple hot repercussiva, as they are usually called, although incorrectly, are the following: spica nardi, absinth, cabbage leaves, fruits of the cypress, both kinds of hoarhound, fumitory, cimolith, white lead, seal earth, all kinds of putty, clay, acacia, and all juices, water, oils, flours or powders, that may be made from these substances and also to the whole substance itself, either alone or in combination with others.

Repercussiva in a wider sense of the word, not actually the genuine ones, but such as, with respect to the hot remedies, must still be designated under this term, are the following: atriplex, mercurialis, malve, violet, cold water, vinegar, radish, squash, cucumbers, the seed of the malve, the so-called four cold seeds, manopilium montanum, both kinds of lavender, myrrhs, incense, mastiche, alum, salt, sulphur, oil of rose, squinantum (a kind of perfumed rush), abrotanum, corn flour, both kinds of aristolochia, all simple bitter medicines which do not exceed the second degree of heat, again all such remedies that can be made separately or mixed from the above mentioned substances, such as oils, waters, juices, powders, flours, and the substances themselves.

Regarding the manner of forming compound remedies from the single ones, and as regards the method of their application, three general rules are here given for the three classes referred to.

(*a*) The surgeon takes from one or several juices of one, or several herbs, or of leaves, or of similar drugs, as above mentioned, and such as may be suitable for his purpose, and adds them to three parts of oil of roses or any similar oil, one part vinegar and one-half part of seal earth or clay, so that a mass having the consistency of honey is obtained.

(*b*) For all compound materia, such as may be composed of blood or other cold and warm humors, the surgeon should apply compound local remedies in accordance to the composition of the materiæ, taking more of the above mentioned cold moist, repercussiva, when bile prevails, and in the same manner is he to proceed with the single materiæ of other types, such as the sanguinous, the phlegmatic, and others that may predominate in the composition, and if such materiæ be present in equal parts he is to prescribe the suitable repercussivum for each materia present.

(*c*) Before applying any local remedy, the surgeon should ascertain whether the materia upon which he proposes to operate is hot or cold, and if it be hot whether of a sanguinous or bilious composition, and if it be the former only styptic repercussiva, either alone or in combination, are to be used continually. When the surgeon is dealing with a bilious condition he should apply moist repercussiva which are devoid of styptic properties, either alone or in combination. If the matter is cold he is to apply simple or compound hot comforting repercussiva. In each of the above mentioned cases the above indicated remedies should be continually applied without confounding those which should be used in one case and those that are indicated in other conditions.

He (the surgeon) must never abandon the usual remedy until the desired effect is obtained unless this effect should be delayed beyond measure; then it is quite permissible or even advised to resort to one of several others (drugs) that have the same power and effect, while the condition of the patient remains the same.

The repercussiva which are composed of the above or similar simple drugs, are salves or quasi-salves, numbering twelve in all.

No. 1. Unguentum de fensivum commune, which has been often mentioned in the foregoing pages, is composed as follows:

Rp. Boli armenici unc. 1, olei ros. drachm 3, aceti drachm ½.

If there is fear of a general affection arising, or that it should increase in extent, such for example, as in erysipelas, then a half drachm of seal earth should be added to the above formula. This unguentum when spread around wounds will prevent the formation of acute abscess, erysipelas and hermes, and prevents the spread of general infection and aborts acute tumors wherever it may be applied.

No. 2 is for the same purpose, and is composed as follows:

Rp. Succorum solatri; sempervivæ ana lb. ½, boli unc, 1, ol. ros. unc. 1, aceti unc. ½, are to be mixed together.

No. 3. The same uses. Rp. Sandali albi, spovii acaciæ, ana drachm 2, camphoræ drachm 1, opii drachm ½, mix with the juice of any suitable herb and white violets, and a little vinegar.

No. 4. Sandali rubei unc. 1, camphoræ drachms 2, solatri, sempervivæ, ana a handful; rub up and mix with two ounces of oil of roses and one drachm of rose water. This unguentum checks all flow of the heating materiæ, and also contributes to the protection of the heart against all poisonous matter.

But all the above local remedies and other similar ones, must be smeared around the site of the affection and not upon it, until a phlebotomy has been done, or until some other evacuation has been made, except in such cases where the last mentioned plaster is to be directly applied to the heart in order to protect this organ from the poisonous matter.

No. 5. Warm bran with strong vinegar.

No. 6. An ointment made of white wax and oil of roses, Galen's cerate, will warm the lower temperature and cools overheated matter, (or regions?), it never harms and usually does much good. If it be dissolved and frequently spread on cold water or snow, it will then repercute the bilious matter most excellently. This ointment is prescribed by Galen (IX. de ingenio, Chap. V.), where he discusses the cure of priapism.

The following preparations are valuable, especially for pains in the joints.

No. 7. Starch and camphor in equal parts are pounded together and mixed with rose water, and then applied.

No. 8. White bread crumbs unc. 1, opii unc. ½, mixed with cow's milk.

No. 9. Ol. ros. unc. 1, ceræ unc. ½, to be melted and washed in rose water, then add croci drachm 1, opii drachms ½.

No. 10. Apply lana succida soaked in plain lukewarm vinegar.

No. 11. Lana succida moistened with vinegar to which has been added a decoction of red roses.

No. 12. Flour mixed with the juice of solatrum and a little vinegar.

(3) In this section are given the explanations of which there are five.

(1) Do not be amazed that I have given so many remedies for a single purpose, for as has been seen the same medicine does not help in each and every example of the same disease; besides, all these remedies are not to be found everywhere, and if they are to be obtained they cannot be procured in all seasons, and even if they are found everywhere and in every season, the poor are unable to purchase the more expensive ones.

(2) If the surgeon repercusses in such matter for a considerable length of time, until the region commences to take on a dark discoloration, he must avoid continuing the use of the purely cooling remedies, and he should add some resolutiva or compound local remedies from the juice of solatrum, coriander and cabbage, mixed with barley, bean or other flour.

(3) If an abscess be not repercussed, nor commences to take on a pale blue color, nor is absorbed, but remains the same, becoming neither larger nor smaller, and if the heat and burning present in the diseased structures decreases in intensity, then the surgeon should add resolutiva to the repercussiva, diminishing the latter continually until he does away with them altogether, using only the resolutiva. And although the latter are now applied for the continued treatment of the abscess, they are not to be applied to the abscess itself, but the surgeon is to spread them around the abscess, more especially at that part through which the humors are conducted from the body to the diseased spot.

(4) The most generally accepted opinion is that no abscess can be repercussed or dissolved, and that it is better if it is drawn out and made to suppurate in order that nature may by its means purge the body of the deleterious humors. If, however, the surgeon endeavors to increase the growth and the suppuration, then some say that he does it for malice. If, on the other hand, he repercusses the materia in a rational way and prevents the formation of an abscess, and if after some time the patient should be ill with some other malady, others, or perchance the same people, will accuse the surgeon because he drove away the abscess for personal motives. What is the surgeon to do since he cannot escape criticism, since there is no middle way to be found and since for all that he must act nolens volens? One may say the surgeon should leave the choice to the patient and then proceed to act. If, however, the patient does not select and forces the surgeon to choose, then he must do so, and if he can prevent the formation of pus according to the existing rules, he is to follow what has been explained in Notabel, Chap. III., doctrine 1, treatise 1, having for title the treatment of head injuries and of fractures of the skull, that is, in that part having the following title: "Which is the Better and Most Healthy

Treatment of Wounds, and Similar Injuries, that, Where as far as is Possible the Formation of Pus is Prevented, or that where it is Fostered?"

(5) Now as opium and other narcotics and stupefactives enter into the composition of certain repercussiva, it should be noted that all of these remedies cool on account of their styptic property, whether given internally or applied externally. If they are given internally, in small quantity and diluted by combining with other remedies, they quiet and narcotise, by blunting the sensibility. But when they are applied in large quantities and undiluted, they have a lethal effect and produce the same symptoms on the member to which they are applied in excess. If given in small amounts they only dull its sensibility, they destroy the composition of the member if given in large quantities and thus actually stop the pain, as it is said that the pain is stopped for good in a dead person, since he no longer feels it. Such remedies are opium, mandrake, solatrum, all kinds of henbane, except the white, and all kinds of poppy except the white one. The action of these remedies is more pronounced if they are dry, and amongst the dry ones the following are the most effective: the bark and root of mandrake, the seed of henbane, and of the white and not of the black kinds of poppy. We must warn our disciples against the strong narcotics, and they must be used in small doses and are to be proscribed if death threatens.

(*Concluded in the March number.*)

